# Body mass index and metabolic parameters in patients with schizophrenia during long-term treatment with paliperidone palmitate

**DOI:** 10.1186/1471-244X-14-52

**Published:** 2014-02-22

**Authors:** Jennifer Kern Sliwa, Dong-Jing Fu, Cynthia A Bossie, Ibrahim Turkoz, Larry Alphs

**Affiliations:** 1 Janssen Scientific Affairs, LLC, NJ, USA; 2 Janssen Research & Development, LLC, NJ, USA

**Keywords:** Body mass index, Metabolic, Paliperidone, Schizophrenia, Weight

## Abstract

**Background:**

There is a strong association between weight gain and metabolic events in patients with schizophrenia receiving many of the second-generation antipsychotic agents. We explored the relationship between body mass index (BMI) and metabolic events in patients with schizophrenia receiving long-acting injectable paliperidone palmitate (PP) in a long-term trial.

**Methods:**

We conducted a post hoc analysis of data from a PP study that included a 33-week open-label transition (TR) and maintenance phase; a variable duration, randomized, double-blind (DB), placebo-controlled phase and a 52-week open-label extension (OLE) phase. Overall, 644 patients received PP continuously from study entry through discontinuation or study completion and were grouped by baseline BMI (kg/m^2^): underweight (BMI <19; n = 29, 4.5%), normal-weight (BMI 19- < 25; n = 229, 35.6%), overweight (BMI 25- < 30; n = 232, 36.0%) and obese (BMI ≥30; n = 154, 23.9%). Metabolic treatment-emergent adverse events (TEAEs) and changes in related laboratory results from TR baseline were analyzed.

**Results:**

PP exposure was similar across BMI groups; overall mean (SD) dose/month was 70.3 (17.17) mg eq. [109.6 (26.78) mg]; median duration of exposure was 204 days (6 to 1009 days). Occurrences of metabolic TEAEs overall by group were 0% (underweight), 14.9% (normal-weight), 14.7% (overweight), and 24.0% (obese). The most common (≥2%) metabolic TEAE were weight gain and elevated blood levels of glucose, lipids, and insulin. Mean BMI and weight increased in normal-weight and overweight groups at DB endpoint, and in underweight, normal-weight and overweight groups at OLE endpoint (p ≤0.05). No consistent trend for increased metabolic-related laboratory values by baseline BMI group was observed. Homeostatic model assessments for insulin resistance indicated preexisting insulin resistance at baseline, with minimal changes at OLE endpoint across baseline BMI groups.

**Conclusion:**

Occurrences of metabolic-related TEAEs trended with greater BMI status in patients with schizophrenia treated with PP; consistent trends in metabolic-related laboratory values were not observed.

**Trial registration:**

This study is registered at ClinicalTrials.gov (NCT 00518323).

## Background

Schizophrenia is a chronic mental illness that requires long-term antipsychotic treatment to both manage disease symptoms and delay relapses [1]. Second generation antipsychotics (SGAs) are generally preferred over typical antipsychotics for schizophrenia treatment as they are associated with fewer extrapyramidal symptoms, lower risk of tardive dyskinesia, and possibly greater improvement in negative symptoms [2]. However, several SGAs are known to be associated with a high risk of metabolic adverse effects such as weight gain, hyperlipidemia and hyperglycemia [2,3]. Glucose dysregulation [4], glucose intolerance [5] and increased cholesterol levels [6] can occur in patients taking SGAs and there is a high prevalence of metabolic syndrome, especially in women (52%) compared with men (36%) with schizophrenia [7]. Additionally, obese and overweight patients with schizophrenia are at a higher risk of metabolic adverse effects than those with normal-weight [8]. These metabolic complications increase the risk for cardiovascular diseases, insulin resistance and diabetes mellitus, and can lead to increased morbidity and mortality, in addition to impairing patient adherence to medication [9]. Several consensus panels have recommended regular monitoring of metabolic biomarkers in patients with schizophrenia [10-12].

Paliperidone palmitate (PP), a long-acting injectable (LAI) administered once-monthly (after an initiation regimen of two injections: 150 milligram equivalents (mg eq.) [234 mg] on day 1, followed by 100 mg eq. [156 mg] one week later), has been shown to be effective for the treatment of schizophrenia [13]. We conducted a post hoc analysis of data from a long-term (up to 3 years) multiphase, recurrence prevention study [14,15] to examine the metabolic effects of extended PP treatment in patients with schizophrenia and the association of pre-treatment BMI status on metabolic events.

## Methods

### Study population

The inclusion and exclusion criteria for patients included in this study are reported in detail elsewhere [14,15]. In brief, men and women, aged 18 to 65 years (inclusive), having BMI ≥15.0 kg/m^2^ with a diagnosis of schizophrenia (Diagnostic and Statistical Manual of Mental Disorders, 4th Edition [DSM-IV], criteria) for at least 1 year before screening, and a Positive and Negative Syndrome Scale (PANSS) total score below 120, at screening and baseline were included. Patients were excluded if they had an active DSM-IV diagnosis other than schizophrenia or significant risk of suicidal or aggressive behavior or a suspected history of substance dependence according to the DSM-IV criteria in the 3 months before screening.

For this post hoc analysis, patients who received PP continuously from study entry through discontinuation or study completion were included. Data were grouped according to patients’ baseline BMI: underweight (BMI <19 kg/m^2^), normal-weight (BMI 19 - <25 kg/m^2^), overweight (BMI 25 - <30 kg/m^2^) and obese (BMI ≥30 kg/m^2^).

The study protocol was approved by an Independent Ethics Committee (Comité Ético Científico Universidad de Ciencias Médicas [UCIMED], Costa Rica; Comité de Ética del Centro de Investigación y Extensión de Ciencias de la Salud del Instituto Tecnológico de Estudios Superiores de Monterrey, Mexico; Comité de Enseñanza, Investigación, Capacitación y Ética, Comité de Ética e Investigación Clínica, Mexico; Clinical Trials Department- National Medicines Agency/The National Ethics Committee for the Clinical Trial Study of Medicines, Romania; Independent Interdisciplinary Committee on Ethical Expertise of Clinical Studies, Russia; Pharma-Ethics, South Africa; Central Ethics Commission of the Ministry of Public Health of Ukraine Kornatskyy, Ukraine) or Institutional Review Board (Korea, Taiwan, Sterling Institutional Review Board, USA) at each study site; ethical standards were followed in accordance with the Declaration of Helsinki and consistent with ICH Good Clinical Practices, along with local regulatory requirements. All participants provided written informed consent.

### Study medication and design

Doses of PP can be expressed both in terms of milligrams of PP and milligram equivalents of the pharmacologically active fraction, paliperidone; 39, 78, 117, 156, and 234 mg doses of PP equate to 25, 50, 75, 100, and 150 mg eq. of paliperidone, respectively.

The study consisted of a 9-week open-label transition (TR) phase, a 24-week open-label maintenance phase, a randomized double-blind (DB), placebo-controlled, relapse prevention phase of variable duration, and an optional 52-weeks open-label extension (OLE) phase. In the open-label TR phase, the eligible patients were switched from their previous antipsychotic to an initial regimen of 50 mg eq. PP on days 1 and 8 in the gluteal muscle, followed by a flexibly-dosed PP (25, 50, or 100 mg eq.) once-monthly, in the gluteal muscle. Patients with stable PANSS score (defined as ≤75 at week 9) entered the maintenance phase and received flexibly-dosed PP for the first 12 weeks, with dose adjustments based on patient’s clinical need, followed by 12 weeks of treatment at the established dose. Patients who were stable on fixed dose PP during the maintenance phase were randomized in a 1:1 ratio to continue to receive PP once-monthly or placebo. In the OLE phase, patients received initial dose of 50 mg eq. followed by flexible-dose of 25, 50, 75, or 100 mg eq. PP once-monthly. Doses were titrated up or down in increments of 25 mg eq. at the investigator’s discretion once-monthly for 12 dosing intervals.

### Data analysis

Only patients who received PP continuously from study entry through discontinuation or study completion in the DB and OLE phases were included in data analyses using intent-to-treat (ITT) analysis set, which included patients who received at least 1 dose of PP during the study.

Metabolic treatment-emergent adverse events (TEAEs) were identified using Medical Dictionary for Regulatory Activities (MedRA) preferred terms. All adverse events that occured between the trial reference start and end date were included. Changes in weight, BMI, glucose, and lipid levels from TR baseline to DB endpoint and OLE endpoint were analyzed using a paired *t*-test; no multiplicity adjustment was incorporated. No between-group comparisons were conducted. The effect of PP on glucose homeostasis was assessed by homeostatic model assessments (HOMA), which measured insulin resistance (HOMA-IR) and β-cell function (HOMA-%β) [16]. The HOMA-IR and HOMA-%β values were summarized descriptively. Unadjusted geometrics means and ranges were provided for HOMA-IR and HOMA-%β. This method was applied across all ethnic groups.

## Results

### Patient disposition, baseline characteristics and demographics

**Figure 1:**
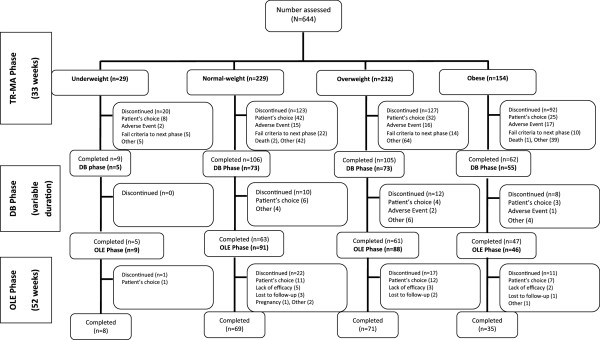
**Patient disposition.** DB- double-blind; OLE- open-label extension; TR-MA- transition and maintenance.

A total of 644 patients met the criteria for inclusion in this analysis, of which 183 completed the study through OLE endpoint. Patient’s choice was the most common (23%) reason for discontinuation, followed by discontinuation due to adverse event (8%) (Figure 1). Only one patient in the overweight group discontinued due to a metabolic-related adverse event (weight gain) during the DB phase. Majority of patients were men (59%) and white (60%). The baseline demographic and clinical characteristics were similar across the groups, except race (Table 1). There was a higher proportion of blacks in the obese group compared with other races. The overall mean (SD) dose of PP during the study was 70.3 (17.17) mg eq. per month. The median duration of PP exposure during the study was 204 days (6 to 1009 days). The mean doses and median duration of PP exposure were similar among all the BMI-based groups.

**Table 1:** Patient demographics and baseline characteristics (Transition baseline)

**Parameter**	**Underweight <19 kg/m**^**2 **^**(n = 29)**	**Normal-weight 19- < 25 kg/m**^**2 **^**(n = 229)**	**Overweight 25- < 30 kg/m**^**2 **^**(n = 232)**	**Obese ≥30 kg/m**^**2 **^**(n = 154)**	**Overall (N = 644)**
**Age (yrs), Mean (SD)**	33.2 (11.20)	36.2 (10.57)	38.2 (10.85)	38.4 (9.81)	37.3 (10.58)
**Men, n (%)**	19 (65)	146 (64)	143 (62)	71 (46)	379 (59)
**Race, n (%)**					
White	19 (66)	147 (64)	139 (60)	82 (53)	387 (60)
Black	3 (10)	30 (13)	37 (16)	50 (32)	120 (19)
Asian	6 (21)	46 (20)	47 (20)	18 (12)	117 (18)
Other	1 (3)	6 (3)	9 (4)	4 (3)	20 (3)
**Baseline BMI (kg/m**^**2**^**), Mean (SD)**	18.0 (0.87)	22.5 (1.52)	27.1 (1.34)	35.4 (4.68)	27.0 (5.88)
**Age at diagnosis of schizophrenia (years), Mean (SD)**	25.4 (8.57)	25.4 (7.39)	26.0 (8.99)	25.4 (8.95)	25.6 (8.41)
**Baseline total PANSS, Mean (SD)**	77.1 (17.20)	71.4 (17.09)	72.6 (18.82)	72.8 (16.91)	72.4 (17.70)
**Prior hospitalization, n (%)**					
None	3 (10)	26 (11)	18 (8)	16 (10)	63 (10)
Once	6 (21)	55 (24)	47 (20)	29 (19)	137 (21)
Twice	9 (31)	49 (21)	48 (21)	40 (26)	146 (23)
Three times	4 (14)	32 (14)	46 (20)	23 (15)	105 (16)
Four times or more	7 (24)	67 (29)	73 (31)	46 (30)	193 (30)

BMI-body metabolic index; PANSS-Positive and Negative Symptom Scale score; SD-standard deviation.

### Treatment-emergent adverse events

**Figure 2:**
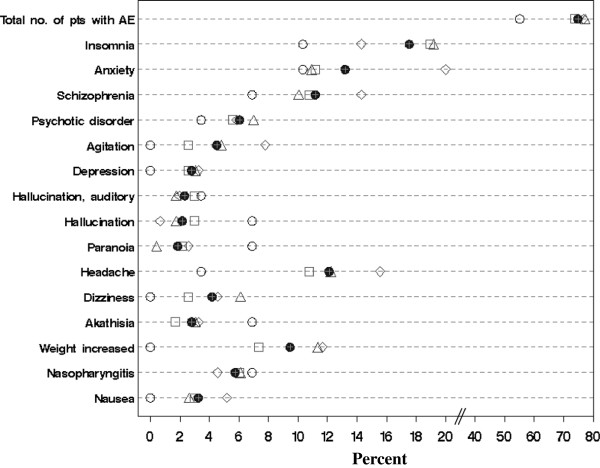
**Treatment-emergent adverse events in ≥5% of patients in any BMI-based group from TR baseline to OLE endpoint. ○** Underweight <19 kg/m^2^ (n = 29) ∆ Normal-weight 19- < 25 kg/m^2^ (n = 229) □ Overweight 25- < 30 ◊ kg/m^2^ (n = 232) Obese ≥30 kg/m^2^ (n = 154) ● Overall (n = 644). BMI- body mass index; TR- transition; OLE- open-label extension.

At least one TEAE from TR baseline to OLE endpoint was reported in more than 70% of patients in each of the BMI groups, except in the underweight group (55%). The most frequently occurring TEAEs (≥5% overall) were insomnia (18%), anxiety (13%), worsening of schizophrenia (11%) and psychotic disorders (6%). Other TEAEs reported in ≥5% of patients in any of the BMI groups were agitation, hallucination, paranoia, headache, dizziness, akathisia, increased weight, nasopharyngitis and nausea (Figure 2, Additional file 1). Two patients in the normal-weight group and one patient in the obese group died during the open-label phase of the study (committed suicide [n = 1], accident [n = 1] and stroke [n = 1]). No clear association of higher baseline BMI with increasing incidence of commonly occurring TEAEs was observed in the study.

**Figure 3:**
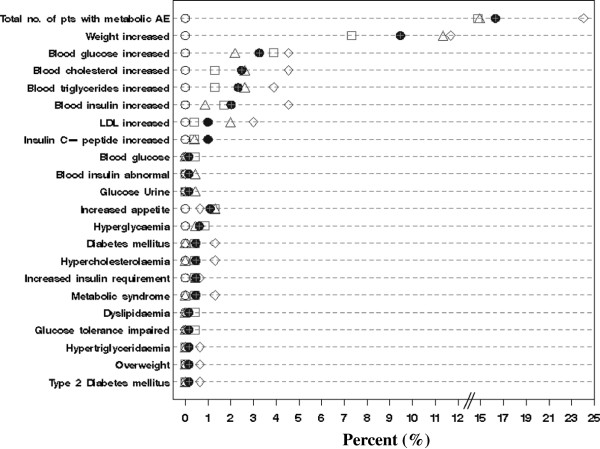
**Metabolic-related adverse events from TR baseline to OLE endpoint.** ○ Underweight <19 kg/m^2^ (n = 29) ∆ Normal-weight 19- < 25 kg/m^2^ (n = 229) □ Overweight 25-◊ <30 kg/m^2^ (n = 232) Obese ≥30 kg/m^2^ (n = 154) ● Overall (n = 644). OLE- open-label extension; TR-transition.

However, the occurrence of metabolic-related TEAEs appeared to differ by BMI status. The obese group experienced the highest overall rate (n = 37/154, 24%) of any metabolic TEAEs and highest rate of most of the specific metabolic-related TEAEs (Figure 3). The overall incidence of metabolic-related TEAEs for the normal-weight (N = 34/229, 14.9%) and the overweight group (N = 34/232, 14.7%) were similar. No metabolic-related TEAEs were reported in the underweight group (N = 29). The most commonly (>2%) occurring metabolic TEAEs by BMI group were weight gain (normal-weight: 11.4%, overweight: 7.3%, obese: 11.7%), elevated blood glucose levels (normal-weight: 2.2%, overweight: 3.9%, obese: 4.6%), and increased levels of cholesterol (normal-weight: 2.6%, overweight: 1.3%, obese: 4.6%), triglyceride (normal-weight: 2.6%, overweight: 1.3%, obese: 3.9%), low density lipoprotein (LDL) (normal-weight: 1.8%, overweight: 0.4%, obese: 2.6%) and insulin (normal-weight: 0.9%, overweight: 1.7%, obese: 4.6%) (Figure 3, Additional file 1).

### Change in mean weight and mean BMI

**Figure 4:**
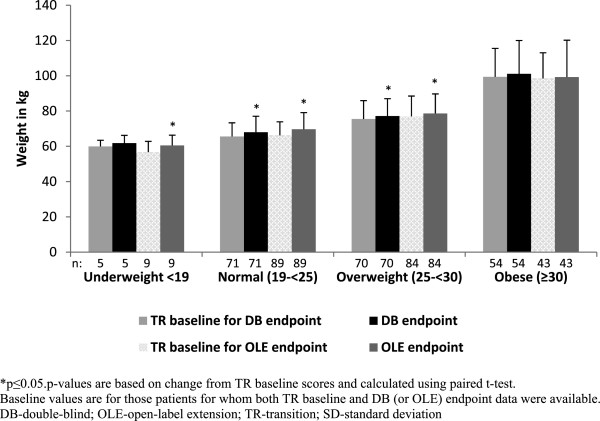
**Mean (SD) weight at TR baseline, DB endpoint and OLE endpoint (ITT analysis set).** *p ≤ 0.05; p values are based on change from TR baseline scores and calculated using paired *t*-test. Baseline values are for those patients for whom both TR baseline and DB (or OLE) endpoint data were available. DB- double-blind; OLE- open-label extension; TR transition; SD- standard deviation.

From TR baseline to DB endpoint (a median duration of 156 days), the mean [SD] weight gain in normal-weight and overweight groups were significant (2.40 [4.99] kg; p <0.001 and 1.68 [5.17] kg; p = 0.008, respectively) (Figure 4).

During this phase, a weight increase of ≥7% was observed in 5% of patients in the normal-weight and obese groups and in 8% of patients in the overweight group. From TR baseline to OLE endpoint (a median duration of 204 days, range = 6 to 1009 days), all BMI-based groups, except the obese group (mean[SD], 0.79 [11.12] kg; p = 0.645), showed significant increases in mean weight (mean[SD], 3.48 [5.99] kg; p < 0.001 for normal-weight group; 1.62 [4.84] kg; p = 0.003 for overweight group); the increase was greatest in the underweight group (mean[SD], 3.82 [4.83] kg; p = 0.0450) (Figure 4). A weight increase of ≥7% from TR baseline to OLE endpoint was observed in 15% of patients in the normal-weight group, 4% of patients in the overweight group, and 6% of patients in the obese group.

**Figure 5:**
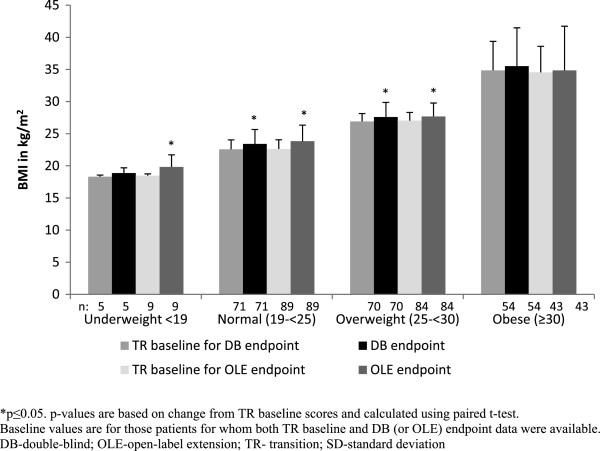
**Mean (SD) BMI at TR baseline, DB endpoint and OLE endpoint (ITT analysis set).** *p ≤ 0.05; p values are based on change from TR baseline scores and calculated using paired *t*-test. Baseline values are for those patients for whom both TR baseline and DB (or OLE) endpoint data were available. DB- double-blind; OLE- open-label extension; TR- transition; SD- standard deviation.

A shift towards a higher BMI category was observed in some patients from TR baseline to DB endpoint (60% [3/5] of patients in underweight group, 18% [13/71] of patients in normal- weight group, and 9% [6/70] of patients in overweight group) as well as from TR baseline to OLE endpoint (67% [6/9] of patients in underweight group, 28% [25/89] of patients in normal-weight group, and 16% [11/70] of patients in overweight group) (Figure 5). However, a few patients from the overweight and obese groups moved to a lower BMI category (TR baseline to DB endpoint: 7% [5/70] moved from overweight to normal-weight group and 9% [5/54] moved from overweight to obese group; TR baseline to OLE endpoint: 9% [8/84] moved from overweight to normal-weight group and 19% [8/43] moved from obese to overweight group).

### Changes in glucose and lipid levels

Mean glucose levels increased significantly from TR baseline to both DB and OLE endpoints in the overweight group, and from TR baseline to OLE endpoint in the normal-weight group (Table 2). An abnormally high (>300 mg/dL; single observation meeting criteria) blood glucose level was observed in one patient each from the overweight and obese groups from TR baseline to DB endpoint. From TR baseline to OLE endpoint, 2 out of 85 patients in the normal-weight group and one out of 41 patients from obese group had abnormally high blood glucose levels (single observation meeting criteria).

**Table 2:** Metabolic parameters at TR baseline, DB endpoint and OLE endpoint (ITT analysis set)

	**Underweight <19 kg/m**^**2**^	**Normal-weight 19- < 25 kg/m**^**2**^	**Overweight 25- < 30 kg/m**^**2**^	**Obese ≥30 kg/m**^**2**^**g**
**Plasma glucose, (mg/dL)**				
ITT analysis set with DB endpoint				
n	5	70	68	52
TR baseline	89.8 (14.65)	92.6 (15.26)	93.8 (12.88)	101.3 (20.02)
Change from TR baseline to DB endpoint	14.6 (18.39)	2.0 (17.89)	7.0 (20.81)	−2.1 (24.20)
p-value	0.1506	0.3563	0.0068	0.5313
ITT analysis set with OLE endpoint				
n	8	85	77	39
TR baseline	90.4 (11.22)	92.88 (19.71)	94.64 (12.01)	100.33. (15.59)
Change from TR baseline to OLE endpoint	3.9 (18.36)	10.2 (29.17)	9.9 (29.00)	3.5 (30.34)
p-value	0.5694	0.0019	0.0036	0.4772
**Insulin, (μU/mL)**				
ITT analysis set with DB endpoint				
n	4	61	63	50
TR baseline	17.0 (13.44)	11.5 (15.23)	12.6 (11.43)	25.6 (32.12)
Change from TR baseline to DB endpoint	−2.5 (4.36)	−0.6 (16.56)	3.2 (21.64)	−4.3 (21.25)
p-value	0.3345	0.7900	0.2517	0.1562
ITT analysis set with OLE endpoint				
n	7	72	76	40
TR baseline	12.3 (11.23)	11.1 (14.16)	14.0 (12.37)	25.0 (34.45)
Change from TR baseline to OLE endpoint	0.3 (17.59)	0.8 (13.71)	1.5 (19.62)	−5.25 (36.13)
p-value	0.9671	0.6288	0.4928	0.3642
**Cholesterol, (mg/dL)**				
ITT analysis set with DB endpoint				
n	5	71	64	52
TR baseline	170.8 (27.09)	180.1 (39.40)	192.7 (34.14)	203.3 (39.23)
Change from TR baseline to DB endpoint	−10.2 (36.27)	−1.7 (28.87)	−8.9 (26.99)	−17.2 (34.78)
p-value	0.5636	0.6320	0.0104	0.0008
ITT analysis set with OLE endpoint				
n	8	85	75	40
TR baseline	161.9 (26.55)	176.6 (34.37)	194.0 (33.87)	199.3 (31.05)
Change from TR baseline to OLE endpoint	−3.25 (28.96)	7.9 (30.87)	−1.0 (36.04)	1.0 (22.43)
p-value	0.7602	0.0202	0.8034	0.7741
**HDL, (mg/dL)**				
ITT analysis set with DB endpoint				
n	5	71	64	51
TR baseline	60.0 (6.44)	55.9 (16.53)	51.0 (12.16)	46.5 (14.16)
Change from TR baseline to DB endpoint	−2.8 (2.86)	−1.6 (12.83)	0.13 (8.93)	−0.2 (9.27)
p-value	0.0941	0.2828	0.9112	0.8805
ITT analysis set with OLE endpoint				
n	8	85	75	39
TR baseline	56.7 (8.38)	53.6 (14.15)	50.7 (12.31)	48.1 (10.75)
Change from TR baseline to OLE endpoint	−0.2 (7.92)	0.3 (13.46)	−1.6 (10.75)	1.0 (10.92)
p-value	0.9314	0.8095	0.1869	0.5514
**LDL, (mg/dL)**				
ITT analysis set with DB endpoint				
n	5	71	61	45
TR baseline	85.4 (18.80)	101.7 (34.37)	113.2 (30.01)	126.3 (35.45)
Change from TR baseline to DB endpoint	−6.0 (23.64)	0.2 (26.18)	−9.6 (23.10)	−18.8 (28.82)
p-value	0.6007	0.9388	0.0020	0.0001
ITT analysis set with OLE endpoint				
n	8	82	72	38
TR baseline	83.2 (20.35)	99.9 (30.97)	113.3 (28.35)	122.9 (28.98)
Change from TR baseline to OLE endpoint	1.7 (21.67)	6.8 (26.86)	−0.1 (30.29)	−2.1 (20.97)
p-value	0.8259	0.0249	0.9814	0.5347
**Triglycerides, (mg/dL)**				
ITT analysis set with DB endpoint				
n	5	71	64	52
TR baseline	124.4 (95.27)	111.4 (57.31)	155.6 (132.87)	172.2 (101.48)
Change from TR baseline to DB endpoint	−5.4 (79.94)	−1.8 (54.93)	−8.5 (129.2)	−0.8 (97.12)
p-value	0.8872	0.7813	0.5985	0.9535
ITT analysis set with OLE endpoint				
n	8	85	75	40
TR baseline	107.7 (77.00)	117.00 (72.70)	158.7 (134.11)	150.5 (67.13)
Change from TR baseline to OLE endpoint	−22.6 (65.95)	5.3 (87.44)	−3.57 (135.8)	13.8 (65.18)
p-value	0.3642	0.5765	0.8203	0.1875

TR- transition; DB- double-blind; OLE- open-label extension; ITT- Intent-to-treat.

Changes from TR baseline to DB endpoint or OLE endpoint were calculated using data from those patients belonging to ITT analysis set with DB or OLE endpoint. All data values are expressed as mean (SD) and p-values are based on change from TR baseline scores and calculated using paired *t*-test.

The decrease in mean (SD) cholesterol (mg/dL) levels from TR baseline to DB endpoint was −10.20 (36.27) for underweight, -1.65 (28.87) for normal-weight, -8.91 (26.99) for overweight, and −17.25 (34.78) for obese groups. However, cholesterol levels increased in the normal-weight and obese groups from TR baseline to OLE endpoint (Table 2). None of the patients showed abnormal (>300 mg/dL) elevation in cholesterol from TR baseline to DB endpoint and only one out of 84 patients in the normal-weight group had an abnormally high cholesterol level from TR baseline to OLE endpoint.

Mean LDL levels decreased in all the BMI groups, except the normal-weight group, from TR baseline to DB endpoint, and the reduction was numerically greater in the overweight and obese groups than in the normal and underweight groups. From TR baseline to OLE endpoint, LDL levels increased significantly (p = 0.024) in patients from the normal-weight group (Table 2). Abnormal increases (>160 mg/dL) in LDL levels from TR baseline to DB endpoint occurred in 4% (2/45) of patients in the normal-weight group and 3% (1/38) of patients in the obese group. In addition, abnormal increases in LDL levels from TR baseline to OLE endpoint occurred in 5% (3/56) of patients in the normal-weight group, 6% (3/53) of patients in the overweight group and 10% (3/30) of patients in the obese group.

There was no significant difference in mean insulin, high density lipoprotein (HDL) or triglyceride levels from baseline to DB and OLE endpoints throughout the study (Table 2). An abnormal increase (>500 mg/dL) in triglyceride levels was observed in 2% (1/51) of patients in the obese group from TR baseline to DB endpoint and in 1% (1/85) of patients in the normal-weight group from TR baseline to OLE endpoint. None of the patients showed an abnormal decrease (<35 mg/dL) in HDL levels throughout the study.

### Homeostatic model assessments

HOMA-IR geometric mean values were >1 in all BMI groups at baseline, indicating preexisting insulin resistance [17]. There was minimal change in the HOMA-IR geometric mean values from TR baseline to OLE endpoint across all baseline BMI groups (Table 3). Baseline geometric mean HOMA-%β values were above normal (defined as >100) in overweight and obese groups, indicating higher β-cell function [17]. At OLE endpoint, HOMA-%β values decreased from TR baseline in the normal-weight, overweight and obese groups. In the underweight group, the HOMA-%β values increased from below to above normal from TR baseline to OLE end point (Table 3).

**Table 3:** HOMA-IR and HOMA-%β values (geometric mean and range) at TR baseline, DB and OLE endpoints

	**Underweight <19 kg/m**^**2**^	**Normal-weight 19- < 25 kg/m**^**2**^	**Overweight 25- < 30 kg/m**^**2**^	**Obese ≥30 kg/m**^**2**^	**Overall**
**HOMA-IR**					
**TR baseline**					
N	22	191	200	133	546
Geometric Mean	1.5	1.7	2.4	4.3	2.4
Range	(0.66, 3.56)	(0.74, 3.99)	(1.11, 5.14)	(1.86, 9.85)	(0.99, 5.85)
**DB endpoint**					
N	4	62	60	52	178
Geometric Mean	2.5	2.0	2.6	3.8	2.6
Range	(0.75, 8.17)	(0.84, 4.64)	(1.13, 5.80)	(1.64, 8.86)	(1.09, 6.33)
**OLE endpoint**					
N	8	76	71	40	195
Geometric Mean	1.9	2.0	2.6	4.2	2.6
Range	(0.73, 4.81)	(0.78, 5.03)	(0.99, 6.97)	(2.19, 8.25)	(1.00, 6.56)
**HOMA-%β**					
**TR baseline**					
N	22	191	200	133	546
Geometric Mean	90.4	93.8	123.4	175.9	120.7
Range	(50.65, 161.25)	(46.18, 190.64)	(63.20, 240.9)	(82.53, 374.68)	(57.47, 53.47)
**DB endpoint**					
N	4	62	60	52	178
Geometric Mean	84.7	100.2	105.9	167.1	118.1
Range	(21.50, 333.65)	(46.54, 215.73)	(59.91, 187.1)	(88.5, 315.3)	(57.8, 241.2)
**OLE endpoint**					
N	8	76	71	40	195
Geometric Mean	117.0	91.0	100.8	167.9	108.2
Range	(50.71, 270.77)	(35.65, 232.06)	(47.68, 213.29)	(91.04, 309.64)	(46.99, 49.21)

TR- transition; DB- double-blind; OLE- open-label extension.

HOMA-IR = Homeostatic Model Assessment for Insulin Resistance; HOMA-%β = Homeostatic Model Assessment for β-cell function. *HOMA-IR and HOMA-%β are expressed as the Geometric Mean (exp [mean {logs} – 1 * SD {logs}], exp [mean {logs} + 1 * SD {logs}]).

## Discussion

Metabolic adverse effects, especially abnormal lipid and glucose metabolism, are a particular concern in patients with schizophrenia receiving SGAs [18-21]. Baseline BMI has been shown to be a potential predictor of increased risk of such metabolic events [8]. Therefore, we conducted a post hoc analysis of data from a long-term multiphase, recurrence prevention trial of PP in patients with schizophrenia [14,15] to assess the occurrence of metabolic events by baseline BMI.

We found no clear pattern, by BMI group, in general TEAEs, but the obese group had the highest occurrence of metabolic-related TEAEs, indicating that higher BMI is potentially associated with higher risk of metabolic-related adverse events. Patients in the underweight group had the lowest incidence of overall TEAEs compared with other baseline BMI groups, and experienced no metabolic-related TEAEs. Adverse changes in metabolic-related laboratory values did not appear to consistently correlate with BMI, however. Reported TEAEs (both overall and metabolic) in this study were consistent with the safety findings of previous studies of PP [22-25].

Weight gain, a common problem of antipsychotic medication, may lead to noncompliance and certain comorbidities such as dyslipidemia, hypertension, diabetes mellitus, cardiovascular disease, cancers, and osteoarthritis [9,26]. A previous meta-analysis demonstrated a mean increase in weight in patients receiving standard doses of antipsychotics over a 10-week period: 4.45 kg with clozapine, 4.15 kg with olanzapine, 2.92 kg with sertindole, 2.10 kg with risperidone, and 0.04 kg with ziprasidone [27]. A double-blind randomized clinical trial comparing three SGAs in patients early in the course of psychotic illness found that at week 12, the olanzapine group had more weight gain, a greater increase in BMI, and a higher proportion of patients with a BMI increase of at least 1 unit compared with the quetiapine and risperidone groups. Furthermore, 80% of patients in the olanzapine group had gained ≥7% of their baseline weight at week 52, compared with 50% in the quetiapine and 58% in the risperidone groups [21]. In the present post hoc analysis, moderate weight gain (0.8-3.8 kg) was observed during the much longer study period (median = 204 days, range = 6 to 1009 days). More patients in the normal-weight group had >7% weight gain compared with all other groups. Even though none of the patients in the underweight group had >7% weight gain, 60% of these patients gained weight and thus achieved normal-weight. More than 50% of patients were followed up to 6 months and 40% of patients were followed up to a year. Although we were unable to follow these patients long enough to determine how the course of their weight gain may continue to change over time, it is possible these patients’ weight trajectory may follow that of other patients with increasing metabolic disturbance occurring during the course of treatment. The weight gain observed in this analysis is comparable to that observed in previous short-term PP studies, where moderate weight gain (0.9-1.5 kg) was observed over a 13-week period [18-20]. Similar findings were also seen in long-term studies with oral paliperidone extended–release, which showed a mean (SD) increase in body weight of 1.2 (5.16) kg from OLE baseline to OLE endpoint [28]. Long-term double-blind, placebo-controlled schizophrenia relapse prevention studies with PP and paliperidone extended-release have shown that increase in body weight is similar between PP and paliperidone extended-release [29]. The mean weight gain observed in the present study is thus parallel to data presented for OLE and short term PP studies. A prospective study examining the use of PP with weight reduction or weight maintenance programs would be valuable.

In addition to their effects on weight gain, SGAs are often associated with abnormal glucose regulation [2,9,16]. In the present study, there were no clinically relevant mean changes in the glucose and serum lipid levels across baseline BMI groups, similar to findings of earlier short-term PP studies [22-25]. Previous long-term PP studies also noted a low proportion of glucose-related TEAEs [24,25]. Further, no clinically relevant lipid changes were observed in a long-term study with the highest available dose of PP (150 mg eq.) [30].

The result of this post hoc analysis should be interpreted cautiously, as the study was not designed or powered to demonstrate differences in metabolic-related TEAEs in subgroups defined by baseline BMI. Additionally, preexisting risk factors in the obese group for metabolic events may contribute to the higher incidence of metabolic-related TEAEs in this group. The results may not be broadly applicable to the full spectrum of patients with schizophrenia due to the specific recruitment criteria for the study, and substantially smaller sample size for the underweight group. Patients with schizophrenia are at increased risk for metabolic abnormalities and schizophrenia treatment seems to exacerbate this risk; these effects could not be assessed in the current study due to lack of a control arm.

## Conclusion

This study, with a median duration of PP exposure of 204 days, suggests that occurrences of metabolic-related TEAEs trend with greater BMI status in patients with schizophrenia treated with PP. Consistent trends in metabolic-related laboratory values with BMI status were not observed. Prespecified studies are needed to confirm these results.

## Competing interests

Drs. Alphs, Bossie, Fu and Sliwa are employees of Janssen Scientific Affairs, LLC. Mr. Turkoz is employee of Janssen Research & Development, LLC. The Janssen companies are Johnson & Johnson companies. All authors hold stocks in Johnson & Johnson. All authors meet ICMJE criteria and all those who fulfilled those criteria are listed as authors. All authors had access to the study data and made the final decision about where to publish these data and approved submission to this journal.

## Authors’ contributions

All authors gave substantial contribution to conception and interpretation of data. Dr. IT was the statistician for this post hoc analysis contributing to analysis of data. All authors reviewed and critically revised the manuscript for important intellectual content. All authors approved the final manuscript.

## Pre-publication history

The pre-publication history for this paper can be accessed here:

http://www.biomedcentral.com/1471-244X/14/52/prepub

## Supplementary Material

Additional file 1 **Supplemental information: appendix 1.** MedRA terminology for TEAEs listed in the manuscript. Click here for file

## Previous presentations

The primary data from this study are published. The data presented in this manuscript were previously presented at the 165^th^ Annual Meeting of the American Psychiatric Association, May 5–9, 2012, Philadelphia, PA, USA.
