# Supplementary material for: Body mass index and metabolic parameters in patients with schizophrenia during long-term treatment with paliperidone palmitate
Source: BMC Psychiatry. 2014 Feb 22;14:52. doi: 10.1186/1471-244X-14-52 (PMC3941932; doi:10.1186/1471-244X-14-52)
Supplement: Additional file 1 — Supplemental information: appendix 1. MedRA terminology for TEAEs listed in the manuscript. [file 1471-244X-14-52-S1.doc]

**Supplemental information:**

**Appendix 1: MedDRA preferred terms for treatment emergent adverse events mentioned in the manuscript.**

Treatment emergent adverse events that may be associated with changes in glucose levels and occurred during each study phase were summarized and mentioned as preferred term.

MedDRA preferred terms defined as related to serum glucose levels are: Diabetic cardiomyopathy, Cataract diabetic, Diabetes complicating pregnancy, Diabetes mellitus, Diabetes mellitus inadequate control, Diabetes mellitus insulin-dependent, Diabetes mellitus non-insulin-dependent, Diabetes with hyperosmolarity, Diabetic amyotrophy, Diabetic autonomic neuropathy, Diabetic blindness, Diabetic bullosis, Diabetic cheiropathy, Diabetic coma, Diabetic complication, Diabetic cystopathy, Diabetic dermopathy, Diabetic end stage renal disease, Diabetic enteropathy, Diabetic eye disease, Diabetic foot, Diabetic foot infection, Diabetic gangrene, Diabetic gastroenteropathy, Diabetic gastroparesis, Diabetic gastropathy, Diabetic glaucoma, Diabetic hyperglycaemic coma, Diabetic hyperosmolar coma, Diabetic ketoacidosis, Diabetic ketoacidotic hyperglycaemic coma, Diabetic macroangiopathy, Diabetic mastopathy, Diabetic microangiopathy, Diabetic nephropathy, Diabetic neuropathic ulcer, Diabetic neuropathy, Diabetic retinal oedema, Diabetic retinopathy, Diabetic ulcer, Diabetic uveitis, Diabetic vascular disorder, Gestational diabetes, Glucose tolerance impaired, Glucose tolerance impaired in pregnancy, Glycosuria during pregnancy, Hyperglycaemia, Hyperglycaemic hyperosmolar nonketotic syndrome, Hypoglycaemia, Hypoglycaemia neonatal, Hypoglycaemic coma, Hypoglycaemic encephalopathy, Hypoglycaemic seizure, Impaired fasting glucose, Impaired insulin secretion, Insulin resistance, Insulin resistance syndrome, Insulin resistant diabetes, Insulin-requiring type II diabetes mellitus, Necrobiosis lipoidica diabeticorum, Neuroglycopenia, Shock hypoglycaemic, Somogyi phenomenon, Blood glucose abnormal, Blood glucose decreased, Blood glucose fluctuation, Blood glucose increased, Blood ketone body, Blood osmolarity increased, Glucose tolerance decreased, Glucose tolerance test abnormal, Glucose urine present, Urine ketone body, Urine ketone body present, Acidosis, Ketoacidosis, Ketosis, Metabolic acidosis, Glycosuria, Ketonuria, Hypoglycaemia unawareness, Increased insulin requirement, Blood osmolarity abnormal, and Hypoglycaemic unconsciousness.
